# On the relationship between external and internal load variables in elite youth soccer players

**DOI:** 10.1038/s41598-025-31487-z

**Published:** 2026-01-22

**Authors:** Nils Haller, Thomas Stanin, Tilmann Strepp, Julia Blumkaitis, Manfred Düring, Thomas Mroz, Wolfgang Trutschnig, Thomas Leonhard Stöggl

**Affiliations:** 1 Department of Sport and Exercise Science, University of Salzburg, Schlossallee 49, Hallein-Rif, Salzburg, 5400 Austria; 2 Department of Sports Medicine, Rehabilitation and Disease Prevention, University of Mainz, Albert-Schweitzer-Straße 22, 55128 Mainz, Germany; 3 Division of Exercise and Movement Science, Institute for Sport Science, University of Göttingen, Sprangerweg 2, 37075 Göttingen, Lower Saxony Germany; 4 Red Bull Data Service GmbH, Am Brunnen 1, Fuschl am See, Salzburg, 5330 Austria; 5 Red Bull Athlete Performance Center, Brunnbachweg 71, Thalgau, Salzburg, 5303 Austria; 6 Working Group for Data Science, Statistics, Stochastics, Department of Artificial Intelligence and Human Interfaces, University of Salzburg, Hellbrunnstraße 34, Salzburg, 5020 Austria

**Keywords:** Biomarkers, Neuromuscular testing, Injury prevention, Football, Load monitoring, Biomarkers, Health care, Medical research, Risk factors

## Abstract

The study investigated the relationship between external and internal training load measures in 25 male elite youth soccer players (age: 16.6 ± 0.9 years, VO_2max_: 59 ± 4 ml/min/kg) over 3 months. External load (i.e., total distance, high metabolic power distance, high-speed running) was quantified using a local positioning system and related to subjective (RPE, sleep quality, drive (energy level)), biochemical (creatine kinase (CK), lactate dehydrogenase (LDH), C-reactive protein (CRP), urea, cortisol, transferrin), and neuromuscular (CMJ) markers. Single day workload (1DL), exponential 7-day workload (7DL), and the acute: chronic workload ratio (ACWR) were calculated. 1DL parameters were correlated (Spearman’s rho) with RPE (range *r* = 0.24 to 0.43, *p* < 0.01) and 1DL distance was negatively related to drive (*r* = − 0.28, *p* < 0.001). LDH correlated positively with training load across all calculation methods (up to *r* = 0.27, *p* < 0.01). CK exhibited positive correlations to ACWR training load (*r* = 0.23 to 0.27, *p* < 0.05), while transferrin (ACWR) and CRP (1DL) showed negative associations to training load (*r* = − 0.21 to − 0.28, *p* < 0.05). CMJ eccentric mean force was negatively correlated with all ACWR training load variables (*r* = − 0.22 to − 0.25, *p* < 0.01). In summary, subjective measures showed stronger and more consistent associations with training load than biomarkers or neuromuscular testing. Practitioners may confidently use well-structured questionnaires for load monitoring in elite youth soccer.

**Supplementary Information:**

The online version contains supplementary material available at 10.1038/s41598-025-31487-z.

## Introduction

In today’s professional soccer, condensed training and competition schedules combined with increasing physical demands pose major challenges for coaches and practitioners^1–3^. To address these challenges, load management involves the systematic planning, monitoring, and adjustment of training and competition loads, aiming to minimize injuries and optimize performance^4,5^. Workload is typically monitored covering both the external (i.e., the physical work) and the internal load (the psychophysiological response)^6^. To monitor the training-related response(s), practitioners usually rely on easy-to-use tools such as questionnaires, heart rate^4,7^, or established biomarkers such as lactate or creatine kinase (CK)^8^. Depending on the resources of the club or athletes, practitioners may also measure a variety of additional metrics such as neuromuscular performance^9^, hamstring strength^10^ or comprehensive biomarker panels^11^. There is, however, no consensus on what variables are the crucial and context-specific ones to monitor both external and internal load^6,12,13^.

Uncertainty also exists on the ideal time points for measuring many of the afore-mentioned parameters, which is particularly relevant considering that biomarkers and other monitoring tools may exhibit distinct temporal dynamics in their response to exercise. One popular example is CK, which does not peak immediately after a training session but rather reaches its maximum level after a variable time delay^14^. This suggests that a delayed CK measurement may provide more valuable insights into training load and fatigue. Despite many uncertainties in selecting appropriate monitoring tools and timing, it is evident that internal load monitoring tools should exhibit a distinct response to training load, ideally remaining unaffected by potential confounding factors like diet or circadian rhythm. Tools should be frequently measurable with results being available without significant delay, and the measurement process should not disrupt the ongoing training regimen^2,15^.

A comprehensive systematic review^7^ examined relationships between acute and chronic external training load and various monitoring tools. Concerning questionnaires, robust associations were found in relation to external load, whereas associations between training load and biomarker concentrations were rather weak. While this may suggest relying on questionnaires, there is consensus that, in addition to subjective tools, the integration of objective measures is crucial in soccer in order to minimize the potential for manipulating or underestimating training load^4,16,17^. Depending on the biomarker used, changes (i.e., increases or decreases) can be expected either based on acute load, chronic load, or both in some cases. Interestingly, some biomarkers remain unchanged despite changes in training load and for certain biomarkers such as TNF-α or iron status, responses to training load are not yet fully elucidated^7^. Particularly in elite soccer, further studies are required to clarify how external load relates to a comprehensive range of internal monitoring tools (including questionnaires, biomarkers, and performance testing) covering the multisystemic exercise response.

Thus, the main issue addressed in this study is the limited understanding of how external training load relates to a broad spectrum of internal load monitoring tools in elite soccer. Building upon the methodology and insights gained from a preliminary four-week study^18^, our objective was to exploratorily assess the relationships between external training load obtained from a local positioning system and various internal training load measures using biomarkers, questionnaires and neuromuscular performance in a standardized elite youth soccer setting over three months. We also assessed the correlations between questionnaires, blood biomarkers and neuromuscular performance to determine possible interdependence. By clarifying the sensitivity and consistency of these tools, the study aims to provide practitioners with evidence to refine monitoring strategies in applied soccer settings.

## Methods

### Ethical approval

The experimental design received approval from the human ethics committee of the Paris-Lodron-University Salzburg (approval GZ 20/2021). All procedures were in accordance with the standards of the Declaration of Helsinki of the World Medical Association. Participants were provided with both verbal and written information about the study and subsequently provided informed written consent.

### Participants and setting

**Fig. 1:**
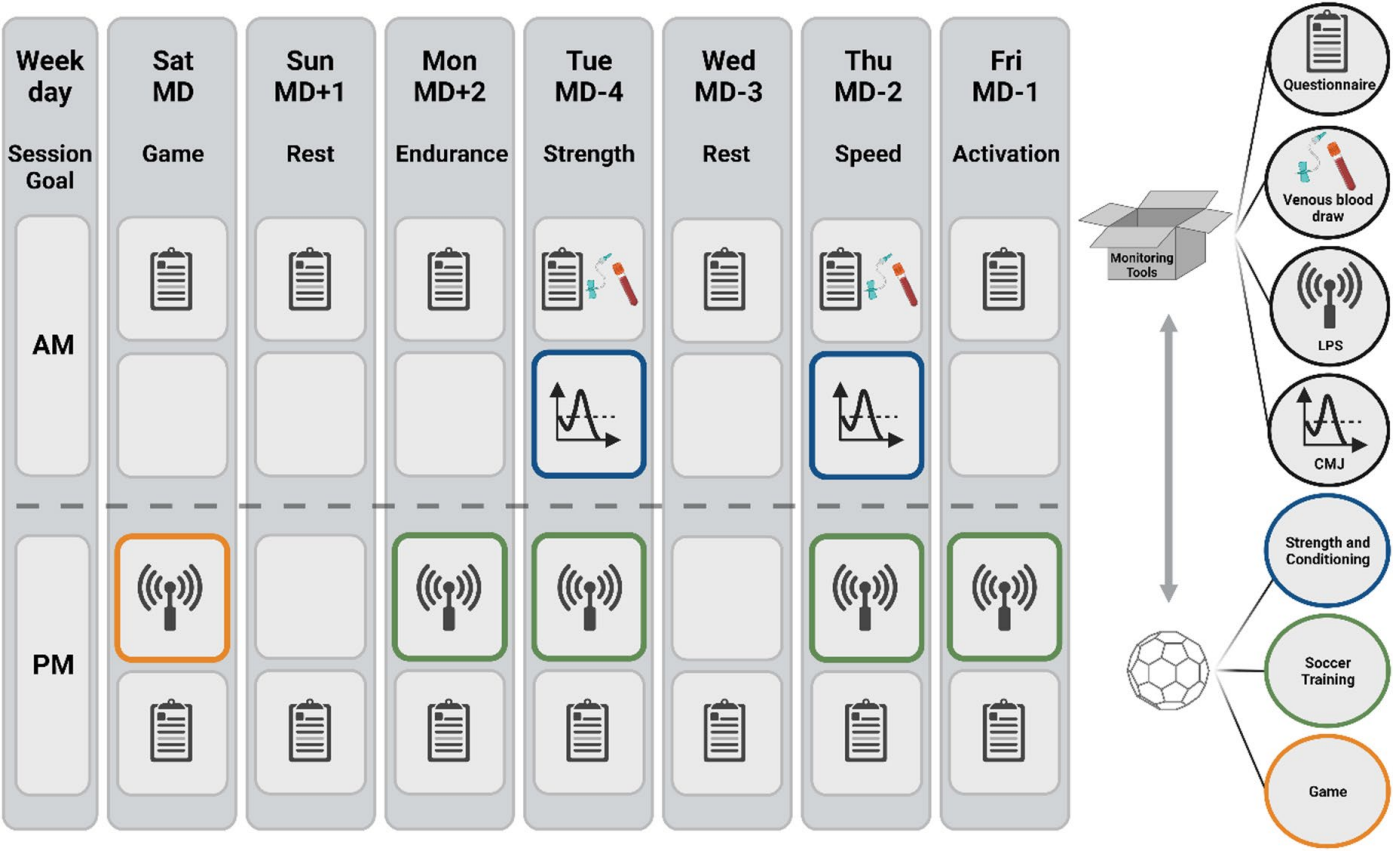
Outline of study design. The legend on the right indicates which measurements were carried out. MD = match day with “+”/“-” signs indicate the distance in days to the regular season matches, AM = morning, PM = afternoon/evening, CMJ = countermovement jump, LPS = local positioning system. The illustration is analogous to the one used in the pilot study^18^. When the entire squad participated in a midweek match on MD − 3, MD − 2 was scheduled as a rest day. In contrast, when only selected players participated in a midweek match on MD − 3, these players participated in regular training on MD − 2. Created with BioRender.com.

Twenty-five male players (mean age: 16.6 ± 0.9 years, average height: 178 ± 7 cm, typical weight: 74 ± 7 kg, and an average maximum oxygen uptake (VO_2max_) of 59 ± 4 ml/min/kg) of an elite European youth soccer team competing in the first national league and participating in the UEFA Youth League were included. Data collection was carried out over a three-month period, from September to December, during the 2021/2022 regular season. Prior to data collection, the participants underwent a familiarization session to understand the objectives of the study. Throughout the data collection process, the researchers did not influence the training program, and the coaching staff received no preliminary results before study completion. Figure 1 outlines the study design.

A standardized setup was used each week to ensure consistency and comparability of the measurements. The training focus and the number of training sessions per day were largely consistent in all weeks, with small variations when additional matches were scheduled in the middle of the week. During the study period, a total of nine games on weekends took place with 5 additional midweek matches. Of these midweek matches, the full squad participated in two, while in the remaining three only one to two players were involved as they were temporarily promoted to the first team. All tests were integrated into the regular training schedule. In particular, participants were instructed to complete questionnaires both in the morning (AM) and in the evening (PM). Strength and conditioning training sessions were carried out twice a week in the morning. Venous blood samples were collected twice weekly, in a fasted state prior to training, on Match day (MD) -4 and − 2 between 7 and 8 AM. This was followed by countermovement jump (CMJ) testing. All players had previous experience with the study procedures, which included CMJ tests and questionnaires; however, venous blood samples were taken regularly for the first time. The entire team’s soccer training sessions and matches were continuously monitored using a local positioning system.

### Measures

#### Physiological exercise testing prior to season start

Players performed physiological exercise testing prior to the season to determine maximal oxygen uptake, peak running speed and lactate threshold using a 2-phase (submaximal step-wise and maximal ramp) test as described elsewhere^19^.

#### Performance data tracking

Performance data such as distance covered, high metabolic power distance (HMPD), and high-speed running (HSR, i.e., speed ≥ 19.8 km/h) were recorded using a 100 Hz local positioning system (Kinexon Precision Technologies, Munich, Germany) during matches and training.

#### Blood collection

Venous blood (~ 3–5 ml) was collected at rest in a fasted condition in the morning, on days MD -4 and MD -2 by certified medical staff and analyzed for white blood cells (WBC), CK, urea, C − reactive Protein (CRP), cortisol, lactate dehydrogenase (LDH), tumor necrosis factor alpha (TNF-α), and transferrin. WBC were analyzed using whole blood by the Mythic 22 Haematology Analyzer (Orphée, Geneva, Switzerland). Serum CK, urea, transferrin, CRP and LDH were analyzed with the Biolis 24i Premium (Marietta, GA, USA). TNF-α was analyzed in duplicates with a bead-based immunoassay (Human Th Cytokine Panel [12-plex], Cat# 741027 and 741028, BioLegend, San Diego, California). Data acquisition was done by flow cytometry (Cytoflex, Beckman Coulter, California, U.S). An ELISA kit (Cortisol ELISA kit, Cat# ADI-901-071, Enzo Life Sciences, Lausen, Switzerland) was used to determine cortisol.

#### Questionnaires

Building upon a cluster analysis of data from our preliminary study^18^, psychologists designed a questionnaire for the present study. Initially, the questionnaire included 23 items for the morning (AM) and eight for the evening (PM). For the present study, this was reduced to five AM questions (sleep quality, sleep onset, wake time, drive (energy level), and muscular fatigue) and five PM questions (Rating of perceived exertion (RPE), general stress, training/game-related stress, self-satisfaction, and mental strength), eliminating redundancy in the assessment of the same psychological domains (and keeping time expenditure for players low). Items were designed in accordance with previous literature^20–22^; however, except for RPE, the single-item measures should be considered practice-based, exploratory indicators rather than validated psychometric constructs.

Questionnaires were administered to participants daily, both in the morning and evening, using the Trayn smartphone app (Sunnyvale, CA, United States). Participants responded to these questions using a Likert scale ranging from 0 to 10, except for questions about sleep and wake times, where specific times were to be reported. The full questionnaire can be found in Supplement S1. For the present study, sleep quality, muscular fatigue, drive (all AM) and the RPE (PM) were analyzed.

#### Neuromuscular performance

CMJ as a proxy of neuromuscular performance were performed on a split force plate (Forcedecks, VALD Performance, Albion, Australia), with arms fixed at the hip. To save time, the jumps were integrated into the 15-minute team warm-up treadmill running session in which the players rotated to perform the jumps and then continued treadmill running. The order of the players to perform the jumps remained the same throughout the study period. Consequently, each player completed the same warm-up duration before their jump attempts, with a minimum of 5 min of treadmill running ensured in all cases. This procedure provided highly standardized conditions while simultaneously reflecting a real-world team training scenario. Following two warm-up jumps (while waiting for the jumps on the force plate), two maximal jump attempts were performed in a standardized order^9,23,24^. Participants were instructed to jump as high as possible in each trial, with the depth of the CMJ chosen by players themselves^18,25^.

### Statistical analyses

All statistical analyses were performed using R (version 4.2.0). Adherence to the questionnaire was calculated by the number of completed questionnaires performed divided by the total number of scheduled questionnaires (i.e., (completed/scheduled) x 100 to express as percentage).

Several quantities contained in the raw data (e.g. exercise time, distances, number of sprints, accelerations, decelerations, metabolic work, distance per minute, HMPD) were removed if HMPD was zero for a particular training session. In the case of several training sessions on the same day, quantities were aggregated whenever possible, non-aggregated variables (e.g., distance per minute, maximal heart rate) were discarded.

Three calculation methods for workloads were considered: For the single day workload (1DL) the previous day’s load was taken (for PM-questionnaires the current day’s load). The exponential 7-day load (7DL) was calculated by summing up weighted loads of the past seven days (for PM-questionnaires the current day and past six days). The considered weights were exponentially decreasing, assigning more recent days higher weight. More precisely, the weighting followed the exponentially weighted moving average (EWMA) approach from Murray et al.^26^ using a decay constant $\:{\lambda\:}_{a}=2/(N+1)$, where $\:N$ was the length of the time-window in consideration. Thirdly, the (7:21 uncoupled^27^ acute: chronic workload ratio (ACWR) was determined by dividing the 7-day load by an exponentially weighted load sum taken over a neighboring but disjoint 21-day time window. Again, exponential weighting followed the approach by Murray et al.^26^ for each time window separately. In all weighting procedures, weights were never normalized. Days with missing data were removed, thereby assigning them a de facto value of zero in the weighted sum. However, time windows only containing NAs were set to NA in the sum. This procedure was applied in the 7DL and ACWR calculations. Since we had no reliable information about potential individual training sessions on off-days, we refrained from any imputation and restricted the analysis to officially tracked sessions. This approach may lead to underestimation of the true training load due to missing information on unrecorded sessions.

Association between several pairs of quantities was quantified using Spearman’s rho. We aimed for a two-level approach: On intra-player level, correlations were calculated separately for each player across time points, while on inter-player level, data was pooled across all players, treating them as repeated measurements coming from a “global” athlete. This provides a view on individualistic patterns (intra-player) while also taking group tendencies into account (inter-player). A significance test (*p* < 0.05) on whether the correlation is zero was conducted. For each considered pair of variables the correlation mean was calculated and tested (*p* < 0.05) for being zero as well (using a two-sided t-test). Quantifying the extent of asymmetry of the resulting Spearman correlations with respect to 0, asymmetry was labelled as ‘high’ if the asymmetry (given by the absolute value of the difference of the number of positive and negative values) was higher than in 95% of a binomial distribution with success probability 0.5, and as ‘low’ otherwise. On intra-player level, sample sizes for the three different workloads (1DL, 7DL, ACWR) varied depending on the metric system groups (blood tests, jumps, questionnaires). For 1DL, mean sample size (i.e., number of pairwise comparisons per player) varied from 6 (blood), 7 (CMJ) to 12 (questionnaire); for 7DL, mean sample size varied from 12 (blood), 16 (CMJ) to 22 (questionnaires); and for ACWR, mean sample size varied from 10 (blood), 14 (CMJ) to 17 (questionnaire).

## Results

Throughout the study duration, no adverse events in the form of injuries or dropouts were observed among the participants due to the monitoring approach. Qualitative feedback from the coaching staff indicated no significant impairment of the training process. Questionnaire adherence for AM questionnaire over the total study period was 29.8%, while PM questionnaire adherence was 20.5%. Sleep times (mean time ± SD in hours) were distributed as follows: sleep onset: 22:42 ± 00:36, wake time: 07:06 ± 01:00. Details on questionnaire adherence are provided in Supplement S2.

Table 1 outlines descriptive data of all tools used in our study.

**Table 1:** Descriptive data.

Match day reference	MD				MD +1			MD +2				MD -4				MD -3			MD -2				MD -1			
	Min	Max	*N*	Mean ± SD	Min	Max	*N*	Min	Max	*N*	Mean ± SD	Min	Max	*N*	Mean ± SD	Min	Max	*N*	Min	Max	*N*	Mean ± SD	Min	Max	*N*	Mean ± SD
**Training load**																										
Total distance (m)	567	11,938	155	5537 ± 3531				1499	5850	87	3857 ± 1024	1167	10,887	122	4481 ± 1232				617	6801	145	3724 ± 1794	1075	6783	135	3420 ± 1192
HSR (m)	0	1170	155	419 ± 258				0	218	87	58 ± 45	0	853	117	117 ± 124				0	446	145	194 ± 89	0	515	135	117 ± 108
HMPD (m)	69	2415	155	1018 ± 573				42	708	87	428 ± 173	61	2687	122	562 ± 359				9	1031	145	440 ± 323	31	1103	135	422 ± 233
**CMJ**																										
Jump height (cm)												27.4	48.7	99	37.8 ± 4.6				28.6	49.7	147	37.9 ± 4.3				
Ecc mean force (N)												598	875	99	714 ± 68				595	881	147	709 ± 69				
Ecc braking RFD (N/s)												1670	9317	99	5202 ± 1419				2368	11,021	147	5177 ± 1468				
**Questionnaires**																										
Sleep (0–10)	5	10	33		5	10	8	4	10	26		4	10	59		6	10	31	3	10	72		6	10	37	
Drive (0–10)	5	10	33		2	9	8	5	10	26		4	10	59		4	9	31	3	10	72		5	10	37	
Muscle (0–10)	2	10	33		1	9	8	2	9	26		2	10	59		3	9	31	2	10	72		2	10	37	
RPE (0–10)	0	10	20		0	6	13	2	8	34		2	9	26		0	6	17	0	8	55		2	7	32	
**Blood**																										
CK (U/L)												64.8	2159.2	71	437.6 ± 351.3				112.4	2164.6	133	440.4 ± 331.7				
CRP (mg/L)												0.0	5.3	71	1.1 ± 1.2				0.0	5.7	114	1.1 ± 1.2				
Urea (mg/dL)												24.7	51.6	71	37.1 ± 6.7				24.0	65.7	113	35.2 ± 6.5				
WBC (10^3^/µL)												3.2	9.4	71	5.7 ± 1.2				3.4	14.0	114	5.7 ± 1.8				
Transferrin (mg/dL)												198	333	71	263 ± 32				197	330	114	256 ± 28				
TNF-α (pg/mL)												4.2	319.2	51	70.2 ± 65.3				3.0	270.1	88	51.4 ± 48.9				
LDH (U/L)												270.7	495.2	71	380.0 ± 55.1				283.7	682.4	114	393.0 ± 64.1				
Cortisol (µg/dL)												18.2	101.8	69	50.3 ± 20.5				17.9	118.3	114	51.6 ± 21.7				

Note that MD statistics include substitutes as well as players who have played the full game. This table only includes ‘regular weeks’ defined as Saturday-to-Saturday match cycles, providing an overview of the typical load within a standard microcycle. In contrast to the subsequent analyses, weeks with additional midweek matches were excluded here to illustrate a representative microcycle.

MD: match day, Min: minimum, Max: maximum, N: sample size, SD: standard deviation, Ecc: eccentric, CK: creatine kinase, WBC: white blood cells, LDH: Lactate Dehydrogenase, TNF-α: Tumor Necrosis Factor Alpha, RPE: Rating of Perceived Exertion.

### Single day workload

**Fig. 2:**
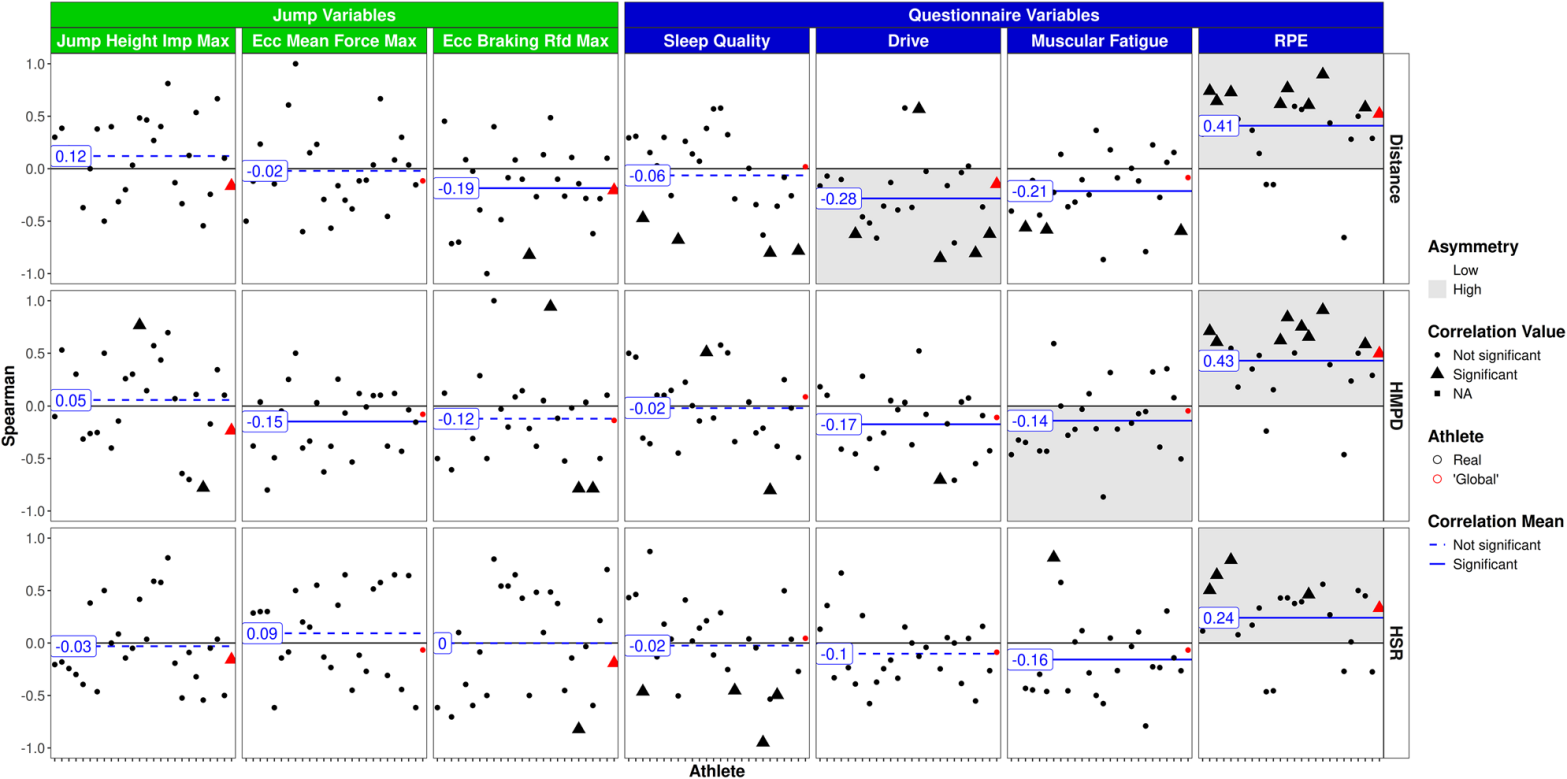
Single day training/game load vs. CMJ and questionnaire variables. Athletes on the x-axis, global player in red. Triangles: significant correlations on individual level; circles: non significant correlations on individual level.

Figure 2 outlines the relationship between 1DL and CMJ as well as questionnaire scores. A pronounced relationship was found between RPE and total distance (*r* = 0.41, *p* < 0.001) as well as RPE and HMPD (*r* = 0.43, *p* < 0.001) with notable asymmetry in both cases. HSR was also significantly correlated with the RPE (*r* = 0.24, *p* < 0.01). In contrast, drive was negatively related to distance covered and HMPD (range: *r* = − 0.17 to − 0.28, *p* < 0.05). No significant correlation was observed between 1DL variables and sleep quality. Furthermore, there were weak, yet noteworthy associations between training load (total distance and HMPD) and CMJ variables, i.e., eccentric mean force and eccentric braking rate of force development (RFD) (range: *r* = − 0.15 to − 0.19, *p* < 0.05).

**Fig. 3:**
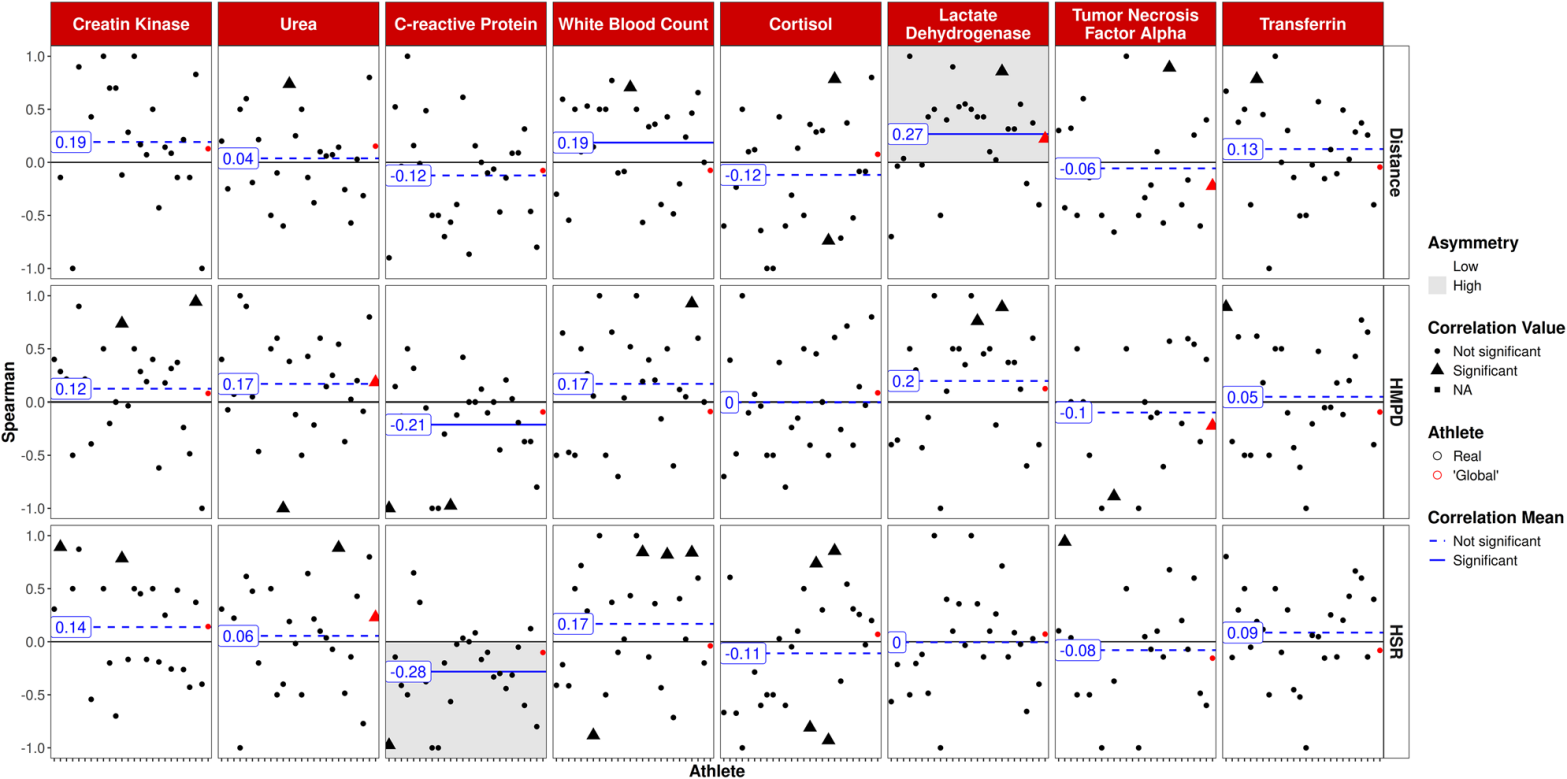
Single day training/game load vs. blood variables. Athletes on the x-axis, global player in red. Triangles: significant correlations on individual level; circles: non significant correlations on individual level.

Figure 3 depicts correlations between 1DL and blood variables, revealing four significant correlations, two of which show high asymmetry. In particular, significant correlations were found between LDH and total distance (*r* = 0.27, *p* < 0.01), WBC and total distance (*r* = 0.19, *p* < 0.05) as well as CRP and HSR (*r* = − 0.28 *p* < 0.01) and CRP and HMPD (*r* = − 0.21, *p* < 0.05).

### 7-day workload

**Fig. 4:**
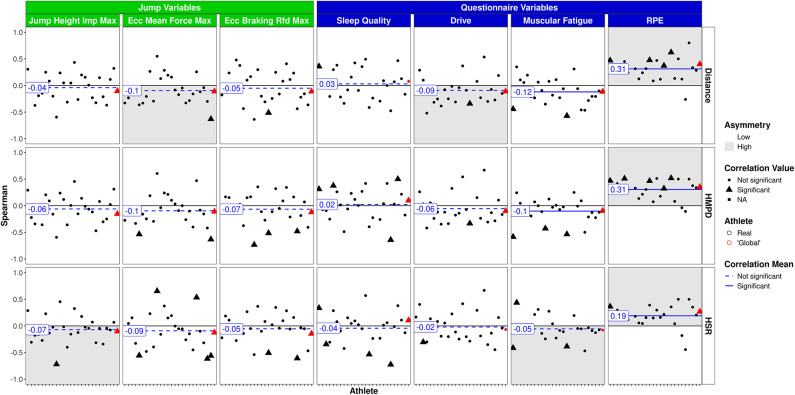
7-day training/game load over the last 7 days vs. CMJ and questionnaire variables. Athletes on the x-axis, global player in red. Triangles: significant correlations on individual level; circles: non significant correlations on individual level.

Figure 4 illustrates the associations between 7DL and questionnaire scores as well as CMJ variables. While tracking variables were again significantly correlated to the RPE (range: *r* = 0.19 – 0.31 *p* < 0.001), no significant associations were observed for CMJ variables.

**Fig. 5:**
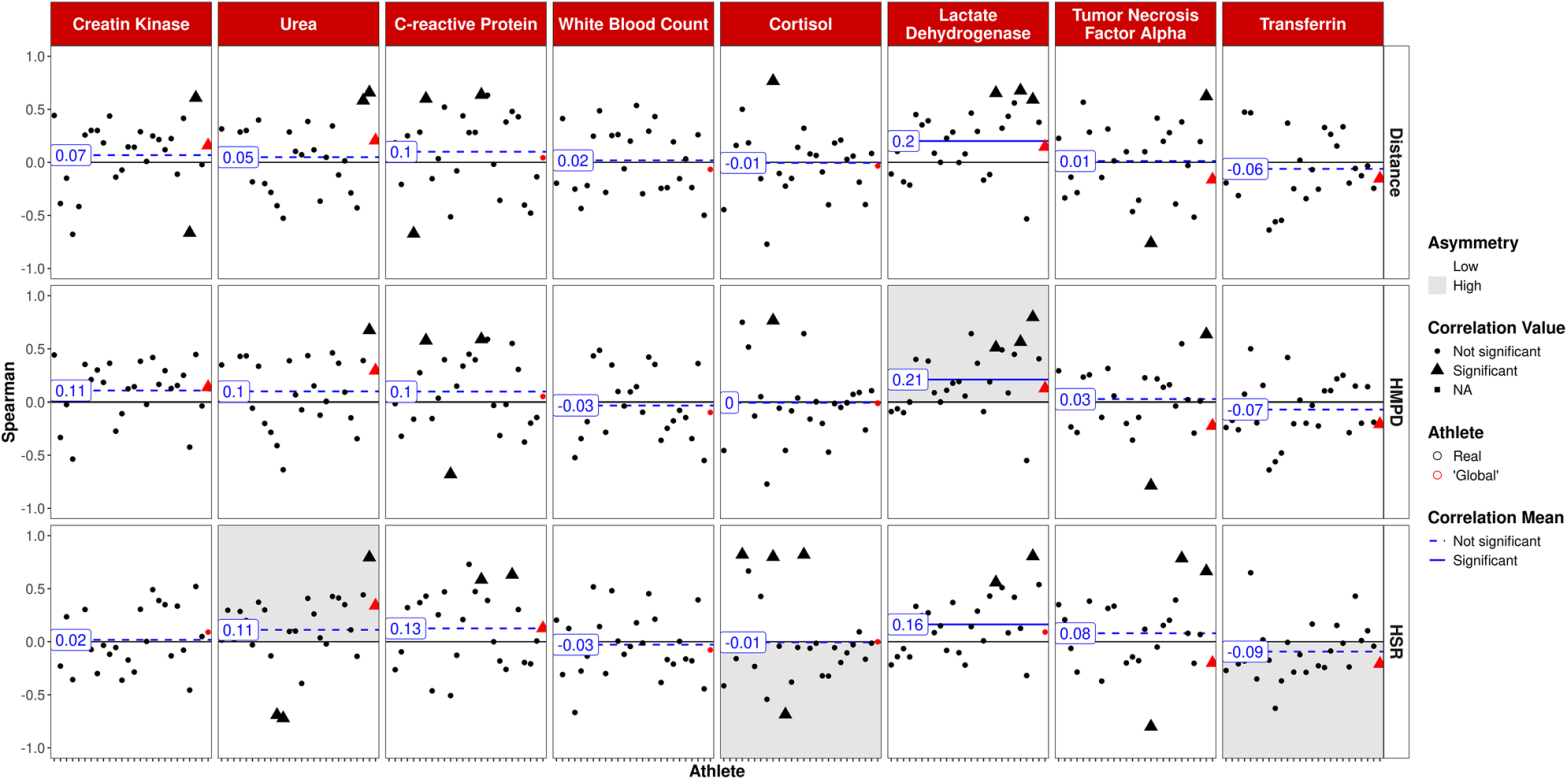
7-day training/game load over the last 7 days vs. blood variables. Athletes on the x-axis, global player in red. Triangles: significant correlations on individual level; circles: non significant correlations on individual level.

Figure 5 outlines the correlations between 7DL and blood parameters. A significant correlation, including high asymmetry, was found between HMPD and LDH concentrations (*r* = 0.21, *p* < 0.01). Additionally, LDH was significantly correlated with both HSR (*r* = 0.16, *p* < 0.01) and total distance (*r* = 0.20, *p* < 0.01).

### Acute: chronic workload ratio

**Fig. 6:**
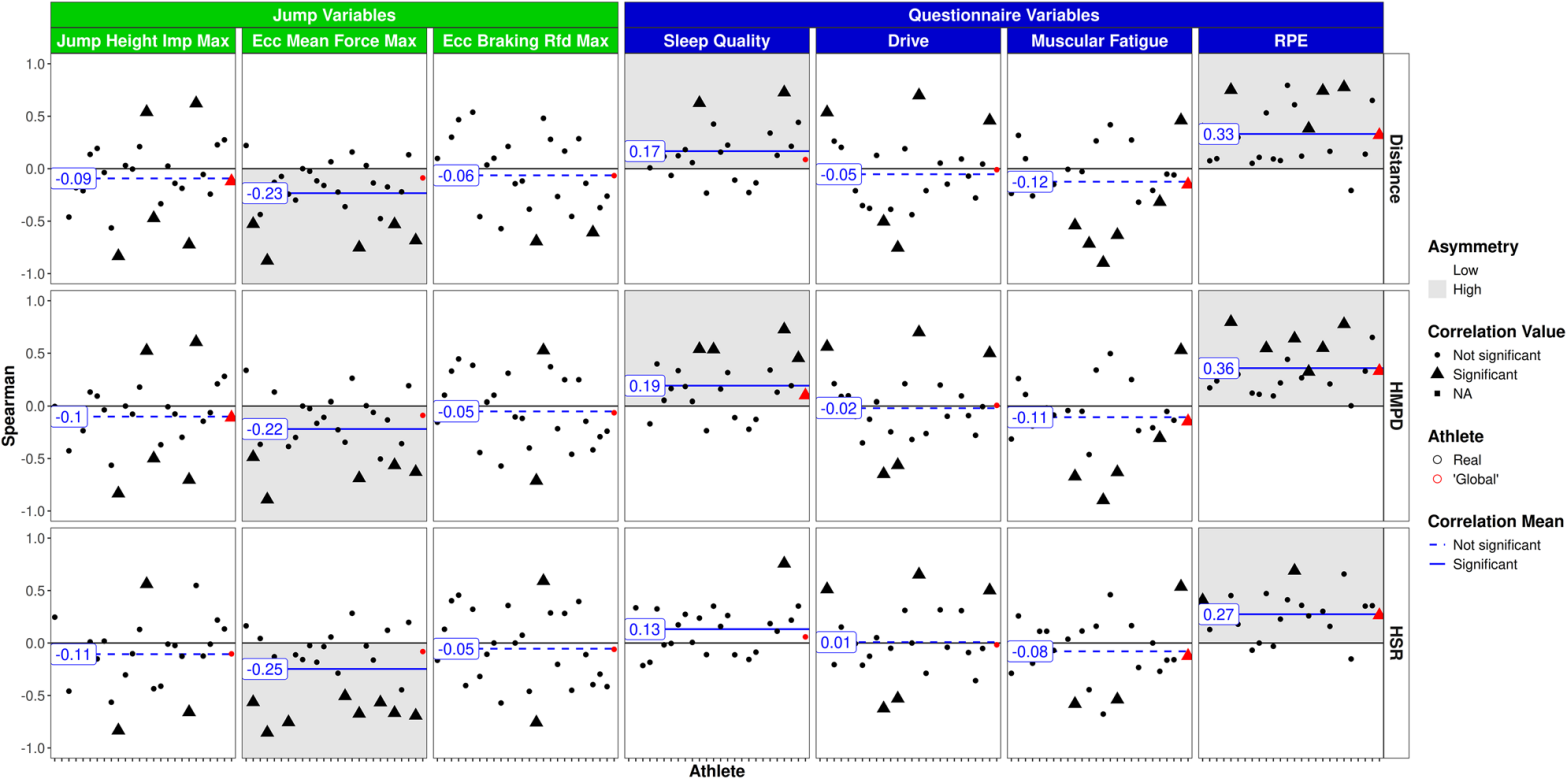
ACWR training/game load vs. CMJ and questionnaire variables. Athletes on the x-axis, global player in red. Triangles: significant correlations on individual level; circles: non significant correlations on individual level.

Figure 6 outlines the ACWR of tracking variables and questionnaire scores as well as CMJ variables. In consistency with findings of 1DL and 7DL, significant associations with high asymmetry were detected between ACWR and the RPE (range *r* = 0.27 to 0.36, *p* < 0.001). Consistent positive relationships were found between ACWR distance and HMPD with sleep quality (range: *r* = 0.17 to 0.19, *p* < 0.01). In contrast, negative relations with notable asymmetry between CMJ eccentric mean force and ACWR tracking data were found (range: *r* = − 0.22 to − 0.25, *p* < 0.01).

**Fig. 7:**
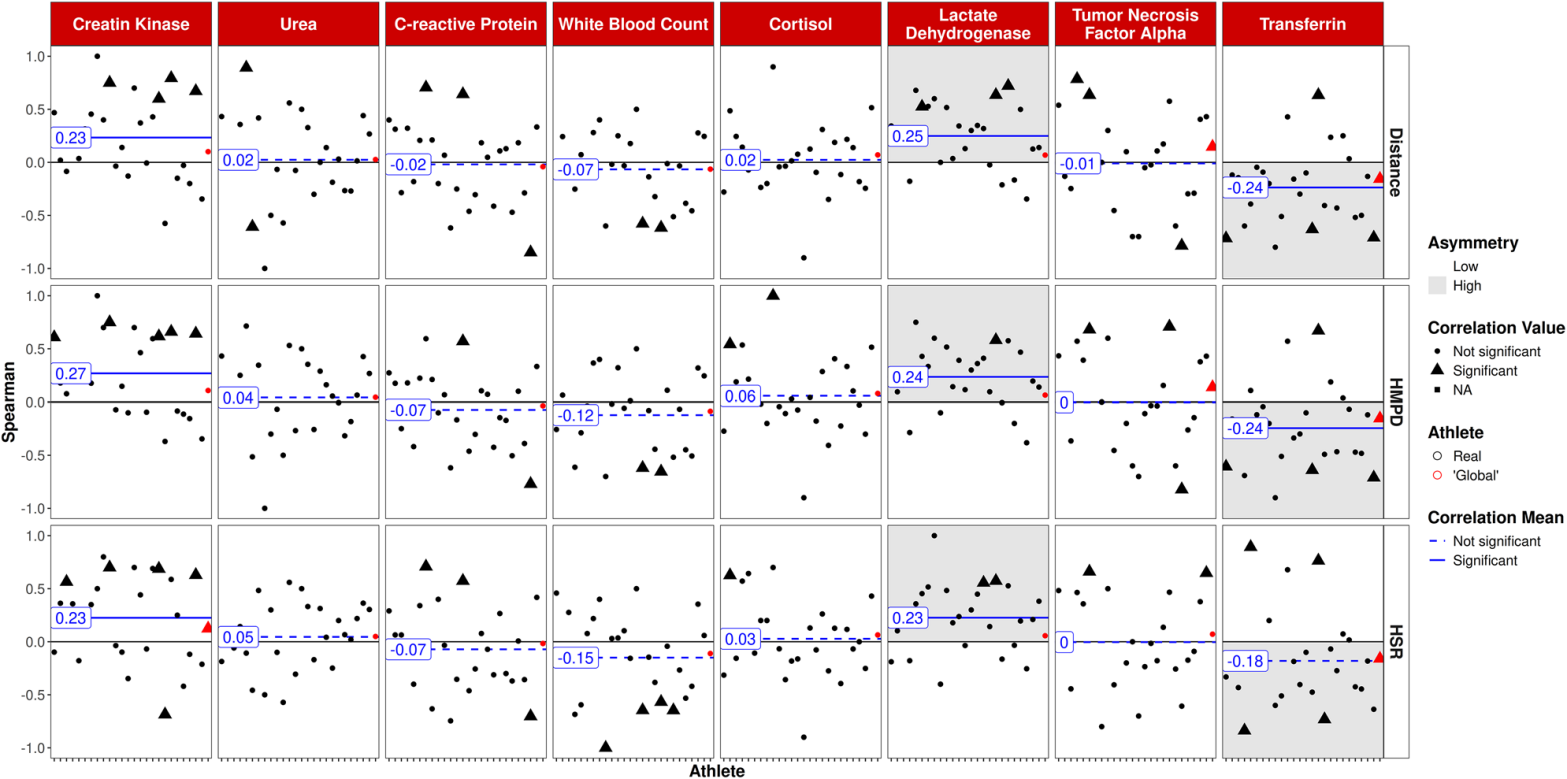
ACWR training/game load vs. blood variables. Athletes on the x-axis, global player in red. Triangles: significant correlations on individual level; circles: non significant correlations on individual level.

LDH was positively correlated with all of the ACWR tracking variables (range *r* = 0.23 to 0.25, *p* < 0.01). Conversely, negative associations were found between transferrin and ACWR tracking variables (range *r* = − 0.18 to − 0.24, *p* = 0.05 (HSR) and *p* < 0.01 (total distance, HMPD). In addition, CK was slightly positively associated with all three tracking parameters (range: *r* = 0.23 to 0.27, *p* < 0.05; Fig. 7).

To demonstrate the robustness of our findings, Figures S3 to S8 excluding all midweek matches (and the corresponding PM/AM measures) are provided in the supplementary material.

### Correlations between blood biomarkers, questionnaire scores and CMJ

**Fig. 8:**
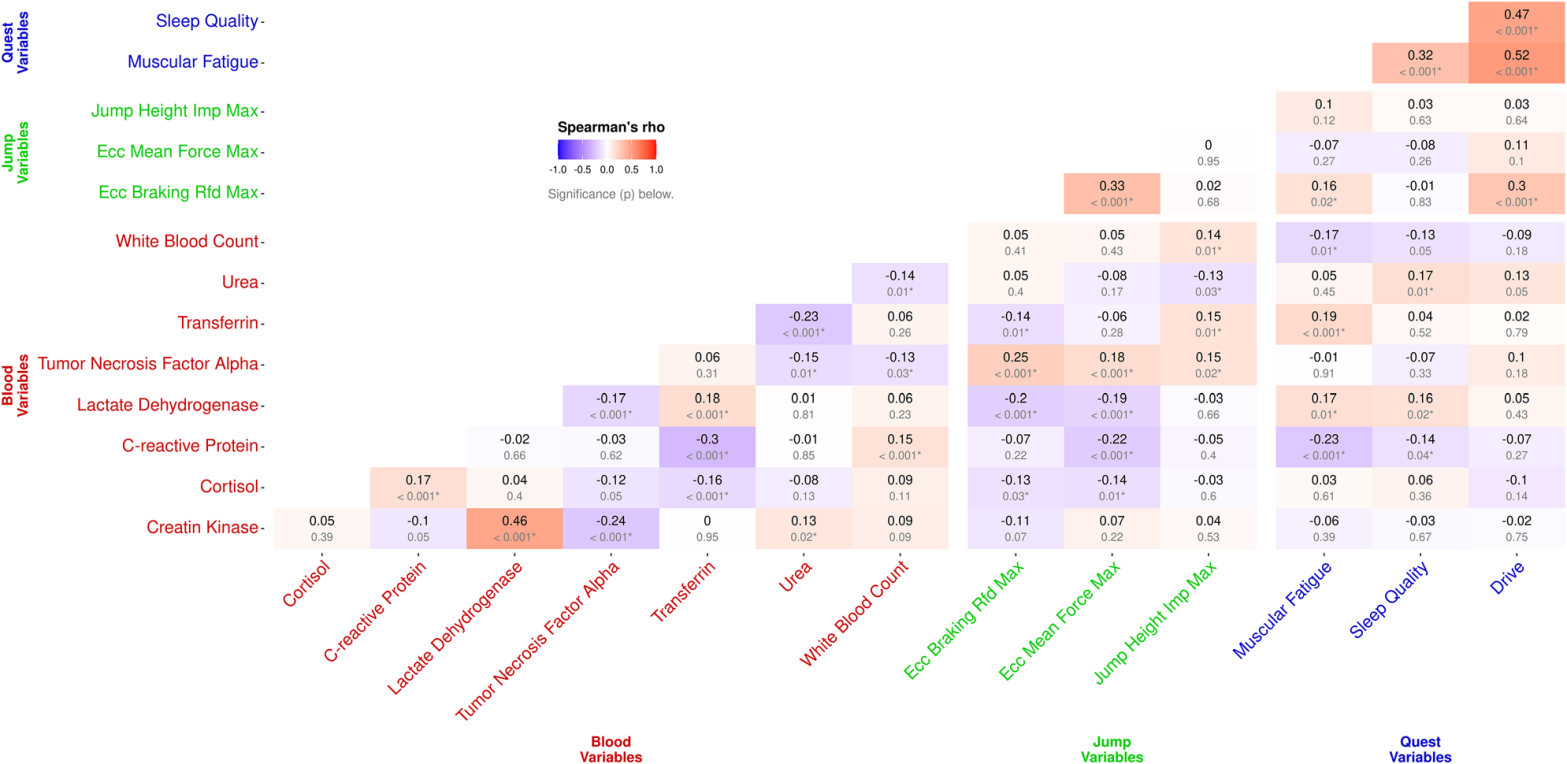
Heatmap with Spearman’s rho between all markers measured at the same time point, i.e., in the morning before regular training.

The correlation heatmap (Fig. 8) provides an overview of relationships among simultaneously assessed markers, i.e., blood, CMJ, and questionnaires. Remarkably, inter-questionnaire correlations ranged from *r* = 0.32 to 0.52 (*p* < 0.001). Biomarkers showed only partial weak correlations with other biomarkers with only one moderate correlation identified between markers of muscle metabolism, namely LDH and CK (*r* = 0.46, *p* < 0.001). With respect to CMJ, eccentric braking RFD and eccentric mean force were significantly correlated with each other (*r* = 0.33, *p* < 0.001). Slight negative correlations were found between eccentric mean force and LDH (*r* = − 0.19, *p* < 0.001) as well as CRP (*r* = − 0.22, *p* < 0.001). CRP was negatively correlated to eccentric braking RFD (*r* = − 0.20, *p* < 0.001). Slightly positive correlations were detected between CMJ variables and TNF-α (range *r* = 0.15 to 0.25, *p* < 0.05). Of note, questionnaire scores were not well reflected by any biomarker.

## Discussion

The main objective of this study was to examine the relationships between external training load quantified using different calculation methods, i.e. 1DL, 7DL, ACWR, and the psychophysiological response in elite soccer players. In addition, relationships between monitoring tools collected simultaneously were explored. We implemented a comprehensive monitoring approach integrated into the daily training regimen. The majority of measures employed were well accepted by players and could be seamlessly integrated into the daily training routine without impacting the training process, as indicated by qualitative feedback from the coaches. Multiple but no consistent associations were observed between external training load and variables collected through monitoring tools, i.e., questionnaires, CMJ and blood parameters, highlighting the complex interplay between objective training load and physiological responses in soccer players. The most robust associations were observed between external training load and questionnaire scores, whereas fewer significant associations were found between training load and blood parameters (particularly LDH) or CMJ metrics. The present findings also suggest that different associations can be found depending on the method chosen to calculate the training load (1DL, 7DL or ACWR).

The RPE proved to be the most sensitive measure for reflecting previous training load across all calculation methods. Assessing the RPE in the evening, rather than the typically suggested 30 min after the session^28^, still resulted in moderate and consistent positive correlations with the external load data. Using 7DL or ACWR instead of 1DL did not improve the correlations with the RPE. In contrast, the ACWR calculation yielded a positive correlation with sleep quality, suggesting that rapid changes in training load (higher ACWR) led to an improvement in sleep quality. Different calculation methods may thus provide added value for monitoring purposes. Interestingly, when using the present data to predict injury risk^25^, sleep quality emerged as the strongest predictor of upcoming injury risk. There is ongoing research regarding the potential relationship between sleep quality and injury risk, with mixed findings to date and an association not yet being fully understood^29,30^. As it is evident from Table 1, adherence to completing questionnaires was relatively high on days when both blood sampling and strength and conditioning sessions took place with lower adherence when players were not under supervision. Implementing education sessions to emphasize the importance of regularly completing questionnaires may improve adherence^31^. In general, the validity of subjective tools in monitoring training and competition load aligns with findings from Saw et al.^7^. Therefore, questionnaires, especially the RPE, should be integrated into the monitoring framework used in (youth) elite soccer as an “easy-to-use” tool^32^.

In contrast to the robust correlations observed with questionnaires, relationships between blood-based biomarkers and training load were comparatively weak and inconsistent among different biomarkers which is in line with findings from a comprehensive systematic review^7^. Few significant correlations, partly including a certain degree of asymmetry (i.e., concentrations are uniformly positively or negatively correlated), were found for LDH, WBC, and CK (positive), as well as transferrin and CRP (negative). Given soccer’s multisystemic physiological response, including muscle damage, inflammation, immune, and metabolic responses^33^, a comprehensive approach utilizing biomarkers in addition to subjective questionnaires is highly warranted^8^. Such an approach may help mitigate the risk of over- or underestimating training load^17^ while potentially uncovering socially desirable responses (e.g., intentional underestimation of training load). In the present study, LDH emerged as the most sensitive biomarker to reflect training load, which aligns with its role as an indicator of muscle damage^34^. In a previous study, increases in LDH were reported immediately after a soccer match in U-21 players^35^. Longitudinal data over a soccer season further support the usefulness of LDH in monitoring both load and recovery status, particularly when analyzed alongside hematological markers^36^. Of note, findings on CK were mixed in our study, with no significant correlations to 1DL and 7DL but consistently with ACWR. CK typically peaks several hours post-exercise depending on the intensity and duration of the exercise^14,33^. Thorpe and Sunderland^37^ revealed acute CK increases even showing a correlation to objective sprint distance in soccer players. Schuth et al.^38^ emphasized that CK changes in youth soccer players are dependent on the player’s position as well as the type of session (training or match), with CK values from one day contributing to the CK values of the next day. Other influencing factors such as muscle mass or impact trauma may have contributed to the inconsistent correlations found in our study^8^. Such a lack of correlation between 1DL training data and CK values might also be due to a so-called repeated bout effect, where constant training loads lead to blunted CK responses due to an increased enzyme inactivation kinetic^39^. A correlation with the ACWR would nevertheless be explainable, as the ACWR serves as a measure of changes in training load. Differences in the correlations between LDH and CK with training load variables could be attributed to the timing of sampling and the interval from the previous training sessions, given that both markers exhibit distinct trajectories and clearance rates depending on the exercise^14,34^.

Negative relationships observed between 1DL and CRP (Fig. 3) as a marker of systemic inflammation may appear somewhat surprising. Typically, CRP levels increase in response to various types of intense exercise even several hours post-exercise^40^. However, chronic training can potentially blunt these responses over the long term. In the context of a regular season in which players were not exposed to excessive training loads (Table 1), it is possible that the lack of a positive correlation between training load and CRP levels reflects the adaptability of athletes to their training program. Transferrin, serving as an indicator of iron status, exhibited no correlation with 1DL or 7DL but demonstrated negative associations with changes in training load (i.e., ACWR). Previous research has shown that various parameters of iron status remain relatively constant throughout a soccer season^41,42^. Instances of iron depletion, iron deficiency, and iron deficiency anemia have been observed in male soccer players at rates of around 5–15%^42^, hence given the absence of a consistent relationship with training load in our study, transferrin is suggested for monitoring iron status in specific players at risk of iron deficiency anemia, particularly when compared with training load and intensity on an individual basis. For a complete picture, further parameters such as transferrin saturation, serum iron, or ferritin should be considered^42^. As iron parameters seem relatively stable, single cross-sectional measurements of the mentioned parameters seem sufficient for certain athletes prone to iron anemia^41^; however, closer monitoring might be relevant in the collective of female soccer players^43^.

It is important to acknowledge that biomarkers may have been influenced by various confounding factors, including environmental conditions, circadian rhythm, and individual clearance dynamics given the time between training and blood sampling^8^. Consequently, biomarkers may fail to reflect the exertion of the preceding day on a group level. Even when biomarkers would have been collected immediately after exercise, individual or cluster-based biomarker responses are expected^44^. We suggest that biomarkers evaluated at rest should be viewed as indicators of general health, fatigue or readiness for upcoming training sessions, and as possible tools for regularly assessing the risk of illness or injury on an individual basis. In terms of their prognostic value, ferritin or CRP may hold promise for predicting or determining illness as shown previously^25^. However, the predictive model in this study exhibited low precision values due to an insufficient sample size and thus needs to be further improved to avoid the risk of false-positive predictions.

Weak correlations were observed for CMJ variables and tracking data. Interestingly, correlation and asymmetry were found to be slightly higher when using ACWR or 1DL compared to the 7DL calculation method. Previous research in soccer and CMJ performance have produced mixed findings. For instance, one study found no effects of training load on CMJ height in youth soccer players^45^, while another study reported a negative relationship between training time and CMJ as well as sprinting performance in male soccer players^46^. These results indicate the complexity of using the CMJ as a proxy for the assessment of training load and fatigue in athletes. Since we have only analyzed three CMJ variables in our study, further research is necessary to identify additional key variables. Future studies should consider incorporating a battery of CMJ variables that reflect both output and movement strategy in their analysis^23^. Due to the numerous variables obtained from CMJ testing, AI-based methods may be suited to detect non-linear patterns between different CMJ variables and to determine clinically relevant changes.

Finally, correlation analyses between monitoring tools collected at the same time point revealed some notable relationships. As expected, significant correlations were found for certain pairs of markers reflecting a similar domain. While questionnaire items as well as certain CMJ metrics correlated with each other, also e.g., biomarkers of muscle metabolism such as CK and LDH were correlated. Interestingly, LDH emerged again as one marker with notable relationships. For instance, LDH was slightly negatively related to CMJ metrics and positively related to muscular fatigue further highlighting LDH as a load and fatigue sensitive measure in soccer.

### Limitations

While correlations between variables provide valuable insights into the interplay of training load and the respective physiological response, they do not necessarily enable definitive conclusions about whether the training load is adequate or excessive or whether there is even an increased risk of injury. Relationships between variables provide an indication of load-sensitive measures for future monitoring approaches. In addition, specific markers showed significant correlations in certain athletes, though these correlations were not apparent at a group level. This suggests the need for an individualized monitoring approach in practice.

While we acknowledge that correlations do not account for confounders or directionality, we chose Spearman correlations due to the ordinal nature of the questionnaire data, which violates key assumptions (e.g., normality) required, e.g., for mixed models. Spearman correlation further enabled consistent comparison across heterogeneous data types (blood markers, jump metrics, questionnaires) without strong assumptions. More complex methods (in particular regression as well as Machine Learning tools like neural networks and random forests) were considered but dismissed due to unmet assumptions, limited interpretability, and the fact that modern nonparametric measures like FOCI are not directly applicable to repeated measures data. Thus, our approach reflects a balance between robustness, interpretability, and data structure. In addition, we applied an exploratory, hypothesis-generating approach; therefore, no formal correction for multiple testing procedures was done. Consequently, individual p-values should be interpreted with caution, and emphasis should be placed on effect sizes and consistent patterns across variables.

Conducting blood sampling twice weekly, limited by organizational constraints, resulted in the omission of potentially crucial sampling time points. A more detailed analysis across multiple time points, including pre-, and post-game/training time points, would have offered a more comprehensive understanding of athletes’ responses to different training loads. In contrast, however, some players were concerned about repeated venous blood sampling. Future research should, thus, prioritize identifying key blood variables and time points for monitoring training load and recovery, preferably measurable via capillary blood or even saliva-based methods. Ideally, this could be achieved through point-of-care testing, similar to the measurement of CK, to minimize discomfort and inconvenience for the athletes^11^. Sampling time points in the morning several hours post-exercise might have been affected by additional activities or recovery strategies aside from the preceding training session^47^. Conversely, for markers with a “time-delayed” response, such as CK, opting for delayed sampling seem more appropriate than immediate post-exercise assessment. The selection of time points in future studies should be tailored depending on the specific marker being studied. Finally, the occurrence of midweek matches disrupted the regular microcycle structure and reduced recovery time between matches, which may have influenced players’ responses and should be considered when interpreting the results. At the same time, any additional matches were fully reflected in the workload calculations, as all training sessions and matches automatically contributed to the accumulated tracking data.

### Practical applications

The approach of our study was feasible with training load-sensitive measures being identified. However, successful integration of such a holistic monitoring approach into the training regime depends on several factors, including coaching staff buy-in, considerations of costs, time, and logistical aspects, team adherence, and having an interdisciplinary team of scientists, physicians, and data analysts^48^. The application of such a concept therefore requires adequate preparation, education for coaches and athletes and the installation of personnel and instruments.

In line with previous research^7^, questionnaires items, especially the RPE, emerge as valuable tools for monitoring training load responses in youth soccer players. Practitioners should thus confidently use well designed questionnaires for monitoring purposes^32^. Considering the weaker associations and the higher burden involved, it is advisable to collect blood samples either when frequent longitudinal data are being collected for certain markers (e.g. LDH via capillary blood) and interpreted in consultation with experienced data analysts. For a comprehensive approach that integrates subjective and objective tools, it is advisable to establish individualized reference ranges at baseline time points^49^ and to define marker-specific “clinically meaningful changes” that require appropriate training load management.

## Conclusions

Various variables (e.g., RPE, drive, CMJ eccentric mean force and braking RFD, LDH, CK, CRP) were associated with prior training load. The most striking correlations were observed with questionnaire items, while blood and CMJ variables showed comparatively weaker associations, often specific to a particular calculation method. Practitioners are advised to confidently apply well designed questionnaires for player monitoring, reserving the use of blood sampling for specific purposes as needed on an individualized basis.

## Supplementary Information

Below is the link to the electronic supplementary material.

Supplementary Material 1

## Data Availability

The dataset used and analyzed during the current study is available from the corresponding author on reasonable request.
