# Supplementary material for: On the relationship between external and internal load variables in elite youth soccer players
Source: Sci Rep. 2026 Jan 22;16:2858. doi: 10.1038/s41598-025-31487-z (PMC12827287; doi:10.1038/s41598-025-31487-z)

**Supplemental Material**

Supplement S1: Questionnaire

Questionnaire items (items in bold, that were analyzed in the current manuscript)

**AM questionnaire** (Likert Scale from 0-10, except sleep onset and wakening):

1. **How was your sleep?** (very poor / very good)
2. **When did you fall asleep**? (exact time)
3. **When did you wake up?** (exact time)
4. **I am feeling** (powerless / full of energy)
5. **My muscular fatigue is** (very fatigued / not at all fatigued)

**PM questionnaire** (Likert Scale from 0-10):

1. **How strenuous was the training / game today?** (rest / maximal)
2. How stressful did you find today compared to an average day? (less stressful / more stressful)
3. How stressful did you find today in terms of training/game compared to an average day? (less stressful / more stressful)
4. Today I was satisfied with myself (not at all / very)
5. I am mentally strong (not at all / very strong)

Supplement S2: Questionnaire adherence

| **Study Week** | **AM_sleep** | **AM_asleep** | **AM_wake** | **AM_drive** | **AM_muscle** | **PM_RPE** | **PM_stress** | **PM_stress** | **PM_satis-faction** | **PM_mental** |
| --- | --- | --- | --- | --- | --- | --- | --- | --- | --- | --- |
| 1 | 90.00% | 90.00% | 90.00% | 90.00% | 90.00% | 76.00% | 76.00% | 76.00% | 76.00% | 76.00% |
| 2 | 62.29% | 62.29% | 62.29% | 62.29% | 62.29% | 44.00% | 44.00% | 44.00% | 44.00% | 44.00% |
| 3 | 30.86% | 30.86% | 30.86% | 30.86% | 30.86% | 12.00% | 12.00% | 12.00% | 12.00% | 12.00% |
| 4 | 28.00% | 28.00% | 28.00% | 28.00% | 28.00% | 10.86% | 10.86% | 10.86% | 10.86% | 10.86% |
| 5 | 48.57% | 48.57% | 48.57% | 48.57% | 48.57% | 36.00% | 36.00% | 36.00% | 36.00% | 36.00% |
| 6 | 37.14% | 37.14% | 37.14% | 37.14% | 37.14% | 25.71% | 25.71% | 25.71% | 25.71% | 25.71% |
| 7 | 30.86% | 30.86% | 30.86% | 30.86% | 30.86% | 27.43% | 27.43% | 27.43% | 27.43% | 27.43% |
| 8 | 27.43% | 27.43% | 27.43% | 27.43% | 27.43% | 17.71% | 17.71% | 17.71% | 17.71% | 17.71% |
| 9 | 16.00% | 16.00% | 16.00% | 16.00% | 16.00% | 11.43% | 11.43% | 11.43% | 11.43% | 11.43% |
| 10 | 9.71% | 9.71% | 9.71% | 9.71% | 9.71% | 4.00% | 4.00% | 4.00% | 4.00% | 4.00% |
| 11 | 13.14% | 13.14% | 13.14% | 13.14% | 13.14% | 8.57% | 8.57% | 8.57% | 8.57% | 8.57% |
| 12 | 12.00% | 12.00% | 12.00% | 12.00% | 12.00% | 10.86% | 10.86% | 10.86% | 10.86% | 10.86% |
| 13 | 6.86% | 6.86% | 6.86% | 6.86% | 6.86% | 5.14% | 5.14% | 5.14% | 5.14% | 5.14% |

Supplement S3: Alternative Version of Figure 2 with midweek games excluded. 1DL vs. CMJ and questionnaires.

**
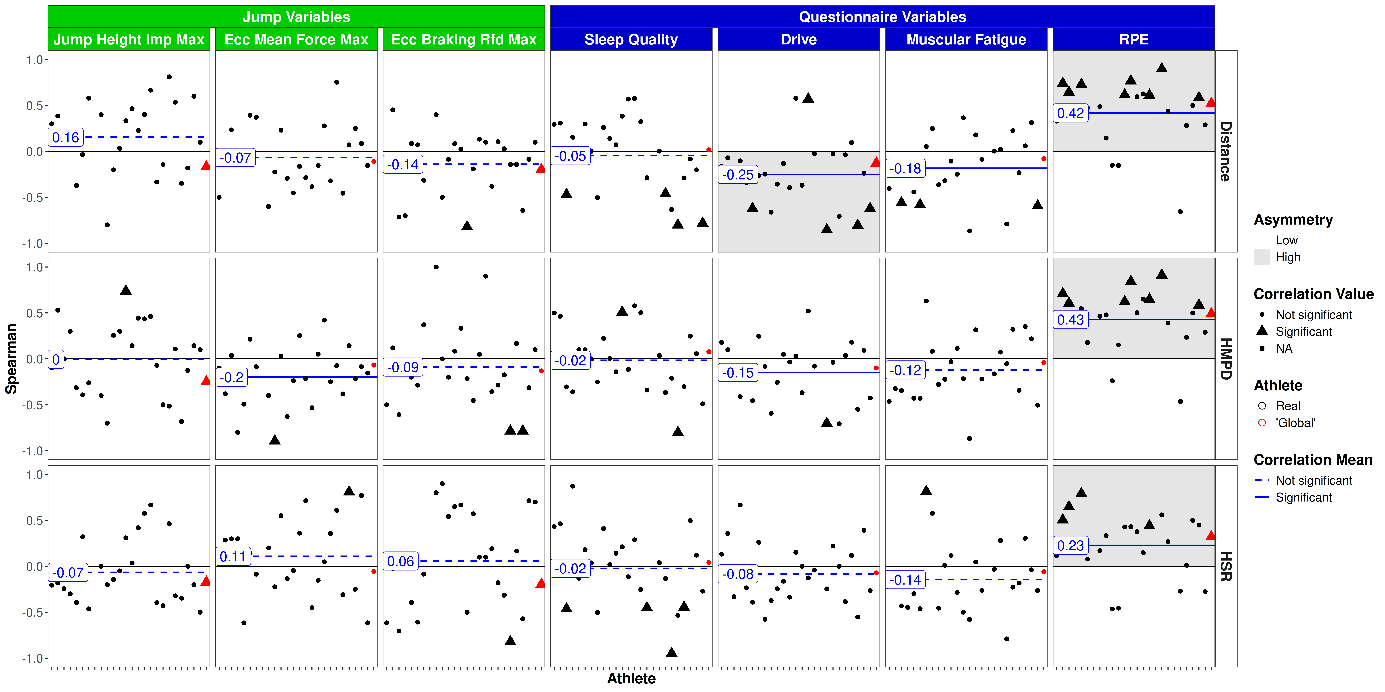
**

Supplement S4: Alternative Version of Figure 3 with midweek games excluded. 1DL vs. blood.


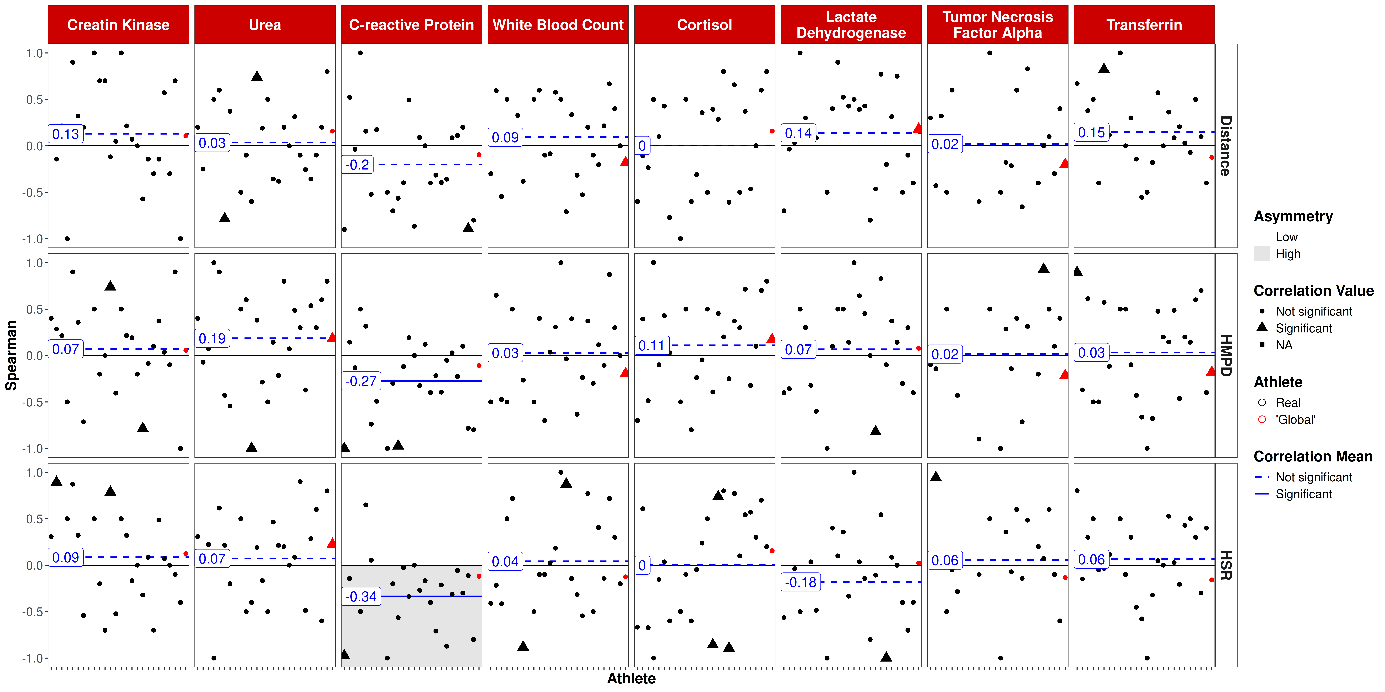


Supplement S5: Alternative Version of Figure 4 with midweek games excluded. 7DL vs. CMJ and questionnaires.


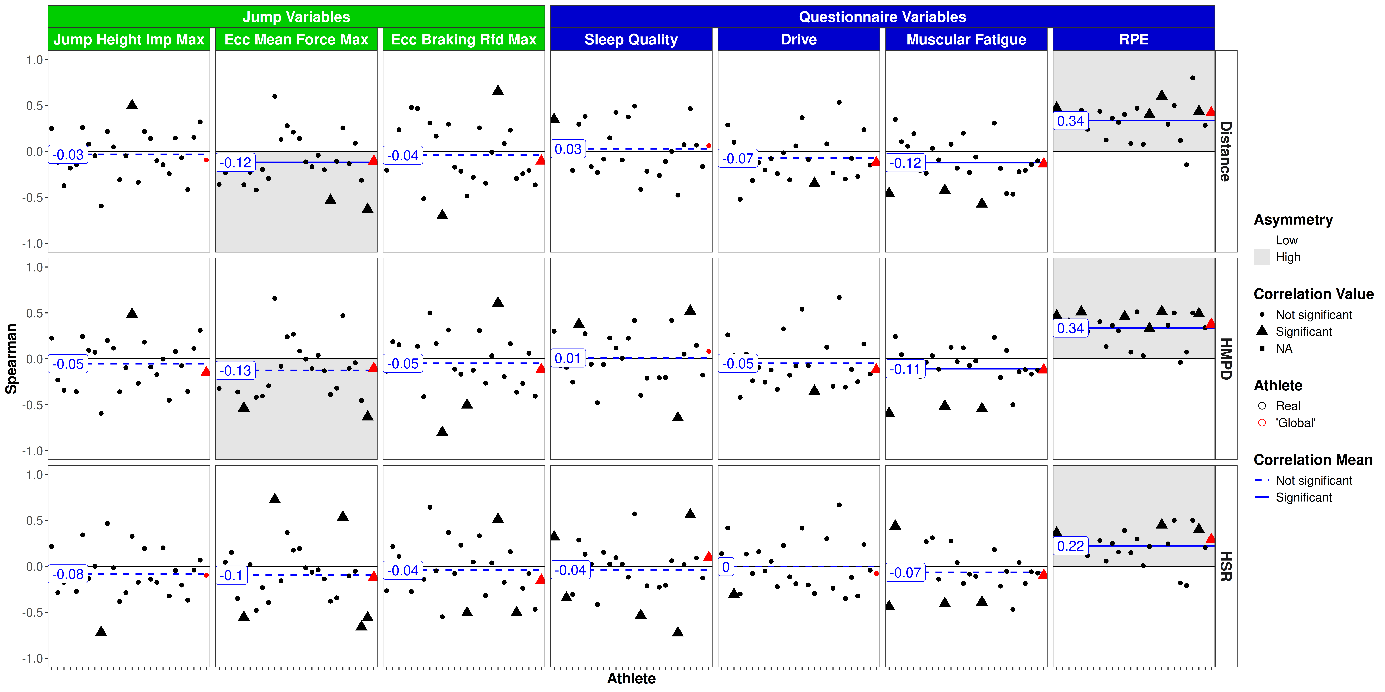


Supplement S6: Alternative Version of Figure 5 with midweek games excluded. 7DL vs. blood.


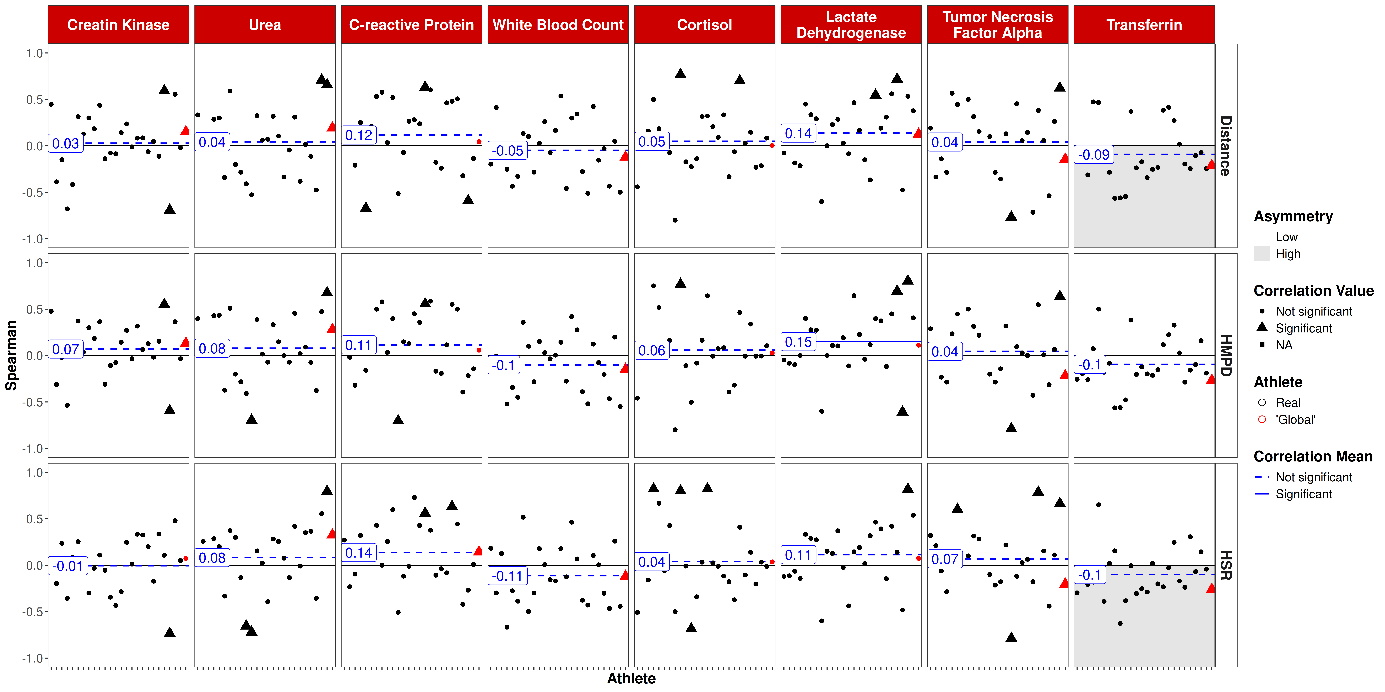


Supplement S7: Alternative Version of Figure 6 with midweek games excluded. ACWR vs. CMJ and questionnaires


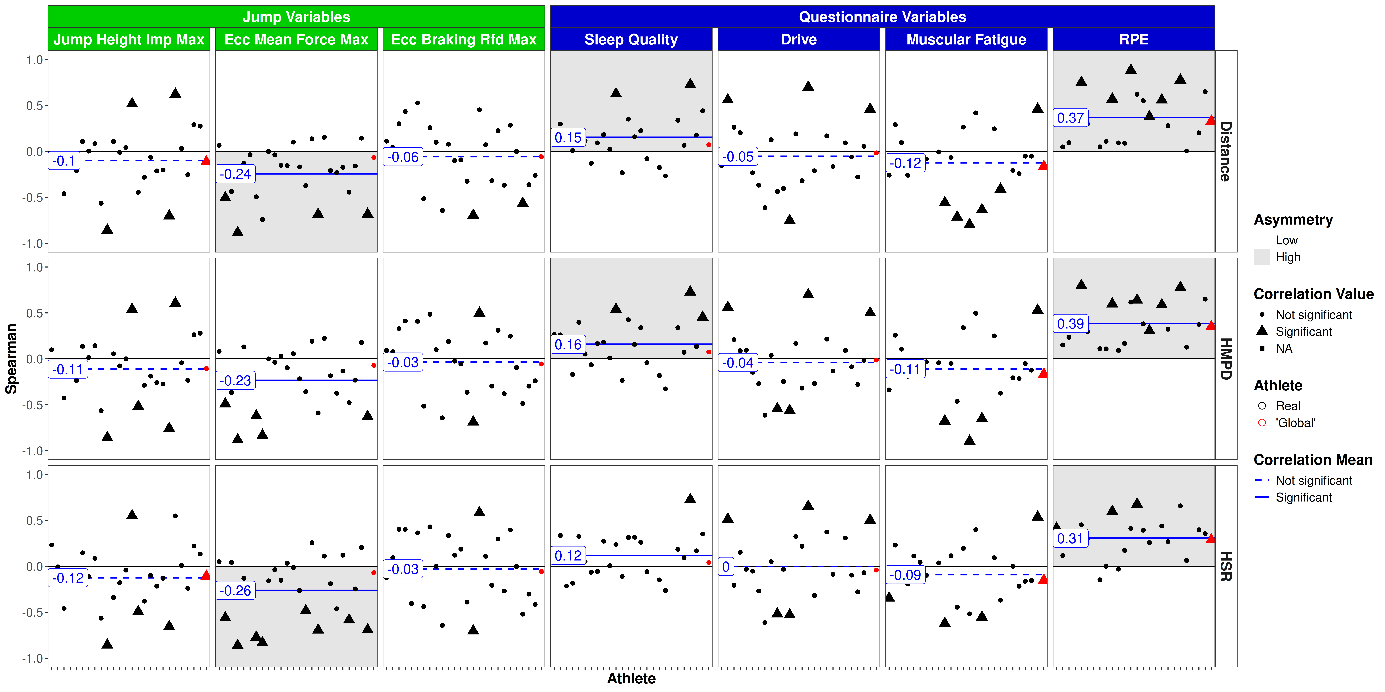


Supplement S8: Alternative Version of Figure 7 with midweek games excluded. ACWR vs. blood.


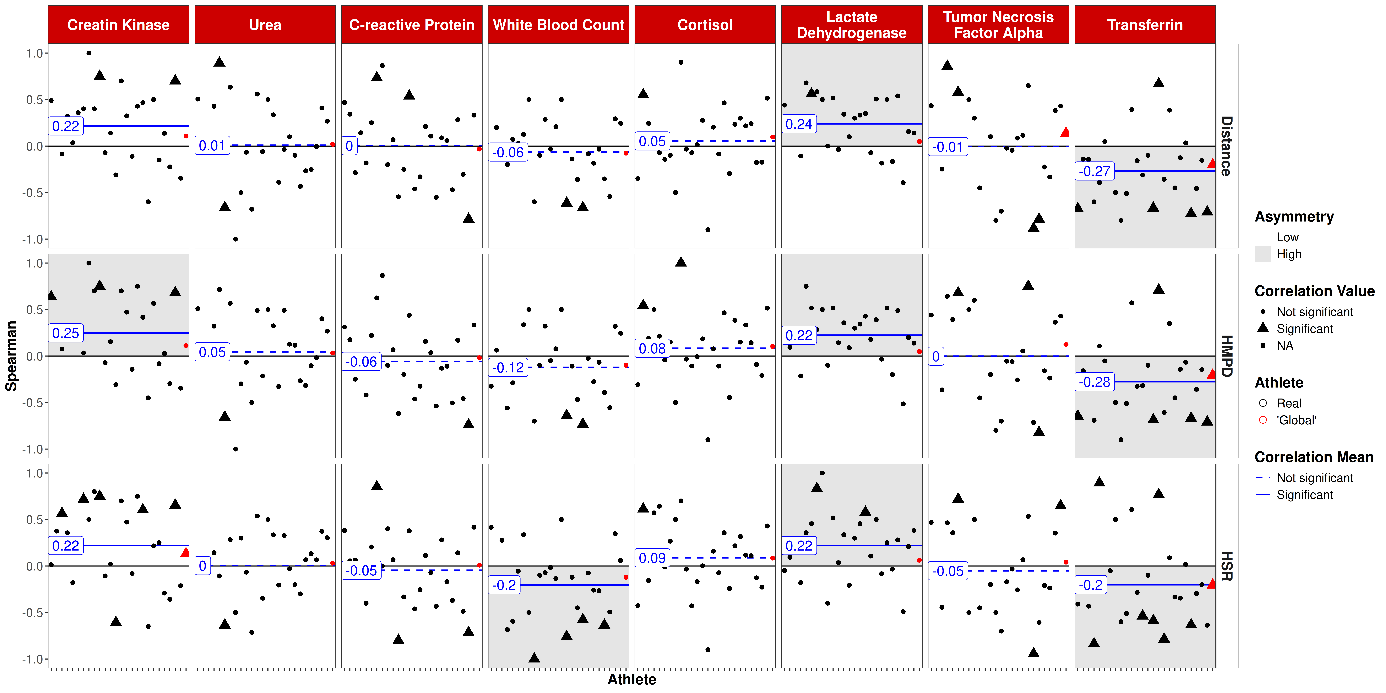

Supplement: Supplementary file 1 — Supplementary Material 1 [file 41598_2025_31487_MOESM1_ESM.docx]
